# Variability in Uremic Control during Continuous Venovenous Hemodiafiltration in Trauma Patients

**DOI:** 10.1155/2012/869237

**Published:** 2012-05-17

**Authors:** Sigrid Beitland, Kjetil Sunde, Harald Moen, Ingrid Os

**Affiliations:** ^1^Division of Emergencies and Critical Care, Department of Anesthesiology, Oslo University Hospital, P.O. Box 4956 Nydalen, 0424 Oslo, Norway; ^2^Institute of Clinical Medicine, Faculty of Medicine, University of Oslo, P.O. Box 1072 Blindern, 0316 Oslo, Norway; ^3^Department of Informatics, Oslo Hospital Services, Oslo University Hospital, P.O. Box 4956 Nydalen, 0424 Oslo, Norway; ^4^Division of Medicine, Department of Nephrology, Oslo University Hospital, P.O. Box 4956 Nydalen, 0424 Oslo, Norway

## Abstract

*Introduction*. Acute kidney injury (AKI) necessitating continuous renal replacement therapy (CRRT) is a severe complication in trauma patients (TP). We wanted to assess daily duration of CRRT and its impact on uremic control in TP. *Material and Methods*. We retrospectively reviewed adult TP, with or without rhabdomyolysis, with AKI undergoing CRRT. Data on daily CRRT duration and causes for temporary stops were collected from the first five CRRT days. Uremic control was assessed by daily changes in serum urea (Δurea) and creatinine (Δcreatinine) concentrations. *Results*. Thirty-six TP were included with a total of 150 CRRT days, 17 (43%) with rhabdomyolysis. The median (interquartile range (IQR)) time per day with CRRT was 19 (15–21) hours. There was a significant correlation between daily CRRT duration and Δurea (*r* = 0.60, *P*≤0.001) and Δcreatinine (*r* = 0.43; *P* = 0.012). CRRT pauses were caused by filter clotting (54%), therapeutic interventions (25%), catheter related problems (10%), filter timeout (6%), and diagnostic procedures (6%). Rhabdomyolysis did not affect the CRRT data. *Conclusions*. TP undergoing CRRT had short daily CRRT duration causing reduced uremic control. Clinicians should modify their daily clinical practice to improve technical skills and achieve sufficient dialysis dose.

## 1. Introduction

Acute kidney injury (AKI) is a serious complication among intensive care unit (ICU) patients associated with increased morbidity and mortality [1, 2]. Dialysis is utilized in the most severe cases to control fluid overload and ensure solute removal [3]. Hemodialysis can be administered either intermittently (IHD) or continuously (CRRT). CRRT is the preferred modality in the ICU because it has a similar mortality [4, 5], but enhanced hemodynamic stability compared to IHD [6]. Several practical aspects regarding CRRT efficacy and outcome are under debate. First, the optimal and appropriate timing of CRRT initiation is questioned [7, 8], as some data suggests that early initiation may convey improved clinical outcomes [8, 9]. Secondly, the optimal dialysis dose is not known, and several studies on mixed ICU patients show conflicting results on how dialysis dose influences dialysis dependency, morbidity, and mortality [10, 11]. Finally, since pauses and interruptions during low-efficacy dialysis such as CRRT may adversely affect uremic control [12, 13], the difference between prescribed and actually performed dialysis dose deserves more attention [12–14]. Uchino et al. showed in a prospective study that temporary stops accounted for 20% of the potential operative time of CRRT, and made the authors suggest that “down-time” from CRRT could serve as a quality indicator, since it was closely related to solute removal [13].

Trauma is a major disease among young people leading to increased disability and mortality [15]. Tissue injury with release of toxic substances and complications such as bleeding and infection might predispose patients to develop organ failures. Kidney function can also be affected; in severe cases CRRT is necessary and contributes to a considerable rise in consumption of healthcare resources [7]. However, in a large multicenter study of AKI in the ICU, trauma patients accounted for only 2% of the patients [16]. Rhabdomyolysis, where striated muscle necrosis leads to release of intracellular muscular constituents such as creatine kinase and myoglobin into the circulation, contributes to development of AKI [17, 18]. Such muscular necrosis might cause kidney toxicity in trauma patients and act together with other prerenal, renal, and postrenal causes of AKI in the ICU [19].

Although clinical data on dialysis therapy is available for mixed ICU populations, data are lacking for the small subgroup of trauma patients. In a recent retrospective study of trauma patients admitted to our surgical ICU, we found an incidence rate of AKI requiring CRRT close to 8%, with a one-year mortality rate of 40% [19]. However, in patients receiving CRRT, we did not report the “down-time,” or possible adverse effects on uremic control. Thus, the aims of this retrospective study were to assess daily duration of CRRT, reasons for temporary stops, and the impact of daily CRRT duration on uremic control in trauma patients with and without rhabdomyolysis admitted to a surgical ICU. Our hypothesis was that trauma patients had several temporary stops during CRRT due to frequent diagnostic procedures and therapeutic interventions causing reduced uremic control.

## 2. Material and Methods

### 2.1. Study Population

Oslo University Hospital is the regional trauma referral centre for approximately 2.5 million people in Norway, whereof 1.93 million adults (>18 years). A retrospective evaluation of all adult trauma patients who developed AKI treated with CRRT between January 1, 1997, and December 31, 2006, was performed. Persons with either chronic renal failure or CRRT lasting less than 24 hours were excluded from further analysis. The study was reviewed and approved by The Norwegian Data Inspectorate and Regional Committee for Medical Research Ethics.

### 2.2. Data Collection

Trauma admissions included all trauma diagnosis codes except late effects, foreign bodies and complications. Diagnosis codes for acute renal failure were used to identify patients with AKI, and procedure codes for continuous dialysis to find patients undergoing CRRT. Information was sought from hospital charts, medical records, institutional trauma, and intensive care registries as well as the national renal registry. Detailed data on daily CRRT duration for each patient was obtained from separate CRRT observational charts. Blood samples at 6 am from the first five days of CRRT were collected.

### 2.3. Study Definitions

The definition of trauma admissions, AKI, and CRRT were as described above and previously presented in detail [19]. Simplified acute physiology score II (SAPS II) [20] was used to assess severity of illness, and the injury severity score (ISS) [21] to assess severity of trauma. Patients were group according to the presence of rhabdomyolysis, which was defined as peak serum creatine kinase above 10.000 U/L (the trigger for initiation of forced alkaline diuresis at our hospital during the study period).

The general indications for dialysis were classified at initiation of CRRT as the follows:

- fluid overload (leading to an oxygenation problem);
- hyperkalemia (serum potassium > 5.0 mmol/L);
- acidosis (whole blood pH < 7.25);
- uremia (serum urea > 30 mmol/L);
- rhabdomyolysis (serum creatine kinase > 10.000 U/L).

CRRT pauses were classified based on the primary cause of the pause as being either:

- filter clotting (dialysis filter pressure primarily increased);
- catheter-related (access and/or return pressure primarily decreased/increased);
- diagnostic procedure (resulting in discontinuation of the CRRT circuit);
- therapeutic intervention (resulting in discontinuation of the CRRT circuit);
- filter timeout (preplanned regular change of filter every 72 hours of CRRT).

Uremic control was assessed by calculating daily percent changes in serum urea and creatinine levels, that is, as Δurea and Δcreatinine. Δurea = (urea_dayN_ − urea_day  N  +  1_)/urea_dayN_, where urea_day N_ is the serum concentration of urea at day N, and urea_day N + 1_ is the serum concentration of urea at day N + 1. Δcreatinine = (creatinine_dayN_–creatinine_dayN+1_)/creatinine_dayN_, where creatinine_day N_ is the serum concentration of creatinine at day N, and creatinine_day N + 1_ is the serum concentration of creatinine at day N + 1. A value of Δurea or Δcreatinine equal to 0 indicated that the serum levels of urea and creatinine did not change from one day to the next; a positive value indicated a decrease, and a negative value an increase in serum concentration. Median values for Δurea and Δcreatinine from the first five days of CRRT were calculated for each individual and were used to assess day-to-day stability in uremic control.

### 2.4. Strategy for Renal Replacement Therapy

The attending intensive care physician prescribed CRRT, while the ICU nurses carried out the treatment. Indication for initiation CRRT was individualized and was frequently consisting of combinations of low urine output, severe hypervolemia, metabolic acidosis, and increased serum concentrations of urea, creatinine, and/or potassium. During the ten years, we used different dual lumen, straight dialysis catheters with length 15–20 centimeters, diameter 11.5–12 French, either Mahurkar (Covidien, MA, USA) or GamCath (Gambro, Lund, Sweden). The dialysis catheters were inserted in the internal jugular, subclavian or femoral vein depending on availability. One CRRT modality was used during the study period, that is, continuous venovenous hemodiafiltration (CVVHDF) using Prisma (Gambro, Lund, Sweden) machines. The dialysis doses were individualized based on body weight and clinical judgment without a specified protocol. Typically settings were blood flow 80–120 mL/minute, dialysate flow: 750–1250 mL/hour and replacement flow: 750–1250 mL/hour. Settings were adjusted to maintain an ultrafiltration rate of 9–14%. Only biocompatible synthetic dialysis membranes were used. Post-dilution mode was the preferred routine, unless recurrent filter clotting indicated the use of the pre-dilution mode. Lactate-buffered solutions were used during the first years, with a gradual change to bicarbonate-buffered fluids currently used. Anticoagulation was often delayed due to increased bleeding tendency, but was otherwise achieved using systemic heparin with target activated prothrombin time (APTT) of 50–70 seconds. Switch to IHD was done in some patients when the hemodynamic situation was stabilized.

### 2.5. Statistical Analyses

Statistical analysis was performed with the statistical package for social sciences (SPSS) for Windows, version 15.0 (SPSS Inc., Chicago, IL, USA). Data are presented for the total population with the subgroups with or without rhabdomyolysis. Categorical data are expressed as number and percent, and compared using Pearson chi-square test, proportions are reported with 95% confidence interval (CI). Continuous data are presented as median and interquartile range (IQR; 25th to 75th percentiles), and compared using Mann-Whitney *U* test. Linear regression analysis was conducted to assess a possible association between the daily CRRT duration and uremic control, and evaluate the property of correlation. The level of statistical significance was set at *P* < 0.05. Based on multiple measurements in each patient, we calculated median values for daily CRRT duration, Δurea, and Δcreatinine for each individual. This was to ensure that calculated *P* values were not invalidated by dependent data set.

## 3. Results

### 3.1. Baseline Patient Data

**Figure 1:**
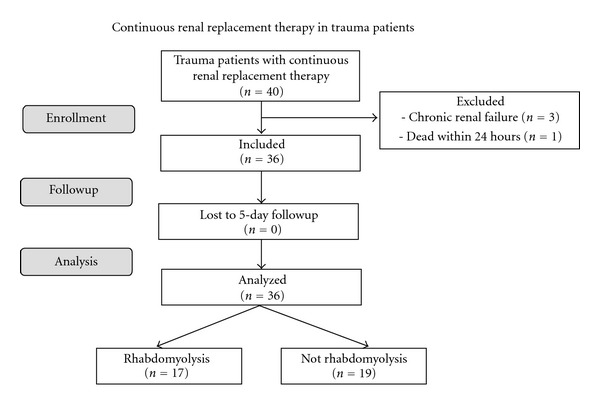
Flow chart of 36 adult trauma patients treated with continuous renal replacement therapy for acute kidney injury grouped with and without rhabdomyolysis.

A total of 40 trauma patients were treated with CRRT during the study period. Four patients were excluded from the analysis due to either known chronic renal failure (*n* = 3) or CRRT lasting less than 24 hours (*n* = 1). Thus, 36 patients with a total of 150 CRRT days were included and further analyzed (Figure 1). Data on median Δurea, and median Δcreatinine are missing for three patients as their CRRT lasted only one day and was terminated before blood samples were drawn at 6 am. Baseline demographics, kidney function, CRRT indications, and performance data as well as outcome are presented in Table 1. The included patients were preferably young males with severe injuries based on their ISS- and SAPS II-scores (Table 1).

### 3.2. CRRT Performance Data

**Figure 2:**
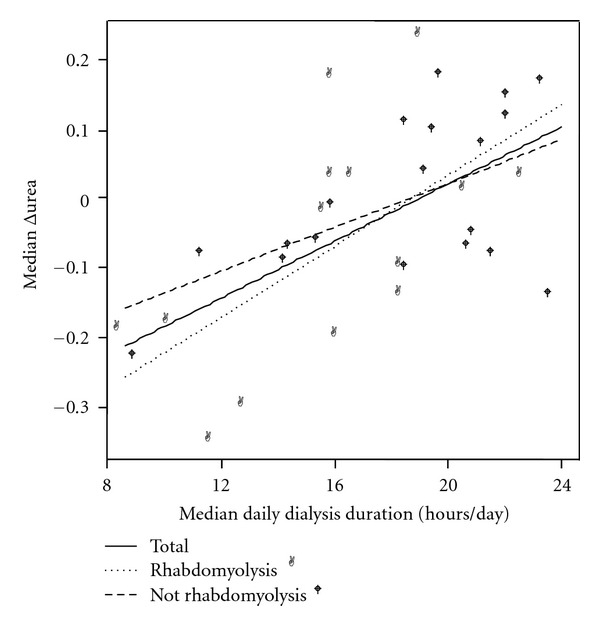
Scattergram of median daily CRRT duration and median Δurea in the total population and the subgroups with and without rhabdomyolysis. Linear regression analysis showed a significant relationship between median daily CRRT duration and median Δurea in the whole group, *r* = 0.60, *P* < 0.001.

**Figure 3:**
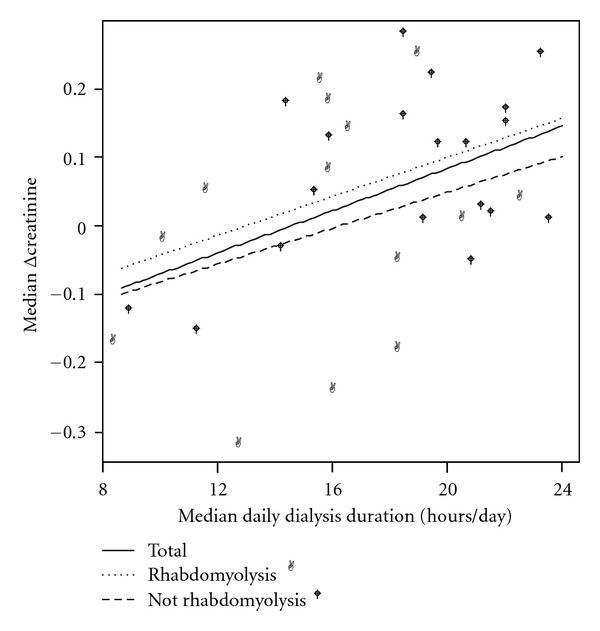
Scattergram of median daily CRRT duration and median Δcreatinine in the total population and the subgroups with and without rhabdomyolysis. Linear regression analysis showed a significant relationship between median daily CRRT duration and median Δcreatinine in the whole group, *r* = 0.43, *P* = 0.012.

Individual data on CRRT performance and uremic control is presented in Table 2. The median daily duration of CRRT was 19 (IQR 15–21) hours (Table 1; Figures 2 and 3), or 78% of the possible operative time. There was a total of 126 temporary stops during CRRT caused by filter clotting 68 (54%, 95% CI 45–63%), therapeutic interventions 32 (25%, 95% CI 17–33%), catheter-related problems 12 (10%, 95% CI 5–15%), filter timeout 7 (6%, 95% CI 2–10%), and diagnostic procedures 7 (6%, 95% CI 2–10%).

### 3.3. Uremic Control during CRRT

Serum concentrations of urea increased during CRRT with median 5.0% per day, whereas serum levels of creatinine decreased with median 5.0% per day. Linear regression analysis showed a significant correlation between median daily CRRT duration and Δurea as well as Δcreatinine. For all patients the median Δurea = −9.36 + 0.49 x median daily CRRT duration, *r* = 0.60, *P* < 0.001. Median Δcreatinine = −5.28 + 0.37 x median daily CRRT duration, *r* = 0.43, *P* = 0.012. With the dialysis dose achieved in these patients, 19.1 and 14.3 hours of CRRT per day were required in order to maintain stable serum concentrations of urea and creatinine, respectively (Figures 2 and 3). 

### 3.4. Rhabdomyolysis

Seventeen (43%) of the patients were classified as having rhabdomyolysis and were compared to the 19 patients without rhabdomyolysis (Tables 1 and 2 and Figures 2 and 3). Reasons for pauses were more often therapeutic interventions in the rhabdomyolysis group (38% versus 12%, resp., *P* < 0.01), and filter timeout in the patients without rhabdomyolysis (2% versus 10%, resp., *P* = 0.04). However, no difference between the two groups was found in median daily CRRT duration (16.3 versus 19.7 hours per day, resp., *P* = 0.19), median Δurea (−0.06 versus −0.05, resp., *P* = 0.20), or median Δcreatinine (0.03 versus 0.12, resp., *P* = 0.21). When comparing patients with and without rhabdomyolysis, no difference in the correlation between median daily CRRT duration and median Δurea (*P* = 0.71), or median Δcreatinine (*P* = 0.36) was found (Figures 2 and 3). 

## 4. Discussion

This study revealed that trauma patients requiring CRRT due to AKI received CRRT median 19 hours per day (78% of total possible time), whereas time on CRRT in other ICU patients in previous studies has varied between19 and 23 hours per day [11, 13, 14].

For several reasons trauma patients with AKI might be different from other ICU patients thereby requiring adjustments of their dialysis therapy. First, trauma patients often have large primary and secondary muscular damage causing rhabdomyolysis and rise in metabolic waste products. Secondly, anticoagulation is challenging since trauma patients often have increased bleeding risk. Finally, as documented in the present study, trauma patients are frequently absent from the ICU due to diagnostic procedures and therapeutic interventions remote from the ICU. However, our study shows that there is a potential for improvement, since almost 2/3 of the CRRT interruptions were due to technical difficulties; either filter clotting or catheter-related problems.

The actually performed CRRT in this study was insufficient in order to reduce serum urea concentrations, but did at least reduce creatinine levels. Above nineteen hours of CRRT per day was required to maintain stable urea levels, and only around 14 hours per day to preserve creatinine concentrations. In contrast, 16 hours of CRRT per day were needed to maintain serum concentrations of both urea and creatinine in a previous study of mixed ICU patients [14]. We might speculate that this difference in dialysis time for maintaining urea and creatinine concentrations in trauma patients could be explained by extensive muscular damage either due to the primary trauma [22] or secondary catabolism [23], which may cause increased production of protein degradation products such as urea. 

Filter clotting (74–78%) was the main reason for interruptions of CRRT in previous studies of mixed ICU patients [13, 14]. Our present data on trauma patients indicates that this filter clotting was responsible for a little more than half of these interruptions. In trauma patients, however, systemic anticoagulation is often restricted due to fair of extensive bleeding, especially in the critical early phase after admission. Some recent data suggest that filter clotting might be counteracted by more use of regional anticoagulation during CRRT [24]. Since filter clotting impact negatively on achieved dialysis dose, it deserves more attention both for trauma patients and mixed ICU patients and warrants clinical studies. Moreover, there is always potential for improvements of technical skills among the clinicians, as catheter-related problems accounted for 10% of the interruptions. Issues such as equipment used, insertion techniques, insertion place, daily routines and hygiene must be a continuous focus in the ICU and deserves more research. 

Diagnostic procedures and therapeutic interventions were in the present study responsible for 31% of the temporary stops during CRRT, while in previous reports of general ICU patients this has occurred more seldom [13, 14]. Trauma patients often have injuries in several organ systems requiring repetitive and longlasting diagnostic procedures and therapeutic interventions such as gastrointestinal, thoracic, orthopedic or brain surgery [25], and/or angiographic embolization [26–28]. This may affect the initial phase of CRRT, as patients often are absent from the ICU during stabilization. A possible handling of this problem might be use of IHD or intraoperative CRRT during prolonged operations, which has been proven to be achievable and safe during other surgical interventions such as liver transplantations [29]. 

Dialysis dosing is a highly relevant clinical issue [30] that during low-efficacy dialysis such as CRRT is dependent not only on the prescribed dialysis doses, but also on the operative time. As CRRT performance data are lacking in trauma patients, this study contributes to new knowledge regarding daily duration of CRRT, reasons for temporary stops and impact of CRRT duration on uremic control in posttraumatic AKI. The clinical implication of our study is that measures should be taken to avoid interruptions during CRRT in trauma patients. This could possibly be achieved by increased use of regional anticoagulation and/or intraoperative use of CRRT. An alternative is to use IHD or increase the prescribed dialysis doses per hour during CRRT in order to achieve adequate solute removal. In our clinical practice, we have now implemented educational programs for CRRT run by dedicated ICU nurses. We have recently also developed a CRRT protocol with standardized indications for CRRT, weight adjusted dialysis doses (approximately 30 mL/kg/hour), and criteria for discontinuation of CRRT. In addition, we have changed anticoagulation from systemic heparin to regional citrate. 

In the subgroup analysis of patients with and without rhabdomyolysis we revealed that median daily CRRT duration and markers for uremic control were similar in the two groups. However, there were more pauses due to therapeutic interventions in the rhabdomyolysis group compared to patients without rhabdomyolysis, as can be expected due to increased need of surgical procedures in this group. 

The main limitation of this study is the small number of patients included, as well as the retrospective and observational design. Uremic control is influenced by a number of factors not accounted for in the present study, such as filter properties, modality of CRRT, gender, body weight, nutritional support and others [31]. It still remains uncertain whether uremic control affects prognosis in general ICU patients and trauma patients. However, uremic control is strongly related to the dialysis dose, but these data were unfortunately not collected and would have added valuable information. Additionally, serum concentrations of urea and creatinine are probably only surrogate markers of uremia, as the relationship between blood levels and toxicity in humans is still under debate [32]. Further, we realize that the present results may not be applicable to other hospitals due to wide variations in the practical performance of CRRT [33–35] and a possible different definition of rhabdomyolysis [17, 18]. The analysis of the two subgroups with and without rhabdomyolysis should be interpreted with caution, as there are relatively few patients in each group with limited statistical power (type II error). Finally, the results of this study cannot be directly compared to former studies of temporary stops during CRRT, since we reported the CRRT duration during the first five days of CRRT, whereas others included all dialysis days in ICU and might even have a different dialysis dose [13, 14]. This study is also stricter in statistical analysis than former studies, since we calculated median values for each person while others calculated data several times from the same individual [13, 14]. Despite these shortcomings, the present retrospective study may serve as a basis for future clinical prospective studies of CRRT and dialysis dosing in trauma patients. 

## 5. Conclusions

Trauma patients undergoing CRRT had relatively short daily CRRT duration causing reduced uremic control. Most of the pauses during CRRT were due to technical difficulties such as filter clotting or catheter-related problems. Clinicians should therefore not only modify their daily clinical practice in order to improve their technical skills and achieve a sufficient dialysis dose, but also keep a continuous focus on quality improvement during CRRT. Future prospective clinical studies of CRRT in trauma patients are needed. 

##  Conflict of Interests

The authors declare that they have no research contracts or conflict of interests.

## Figures and Tables

**Table 1:** Patient characteristics: Trauma patients with acute kidney injury with continuous renal replacement therapy (CRRT) grouped with and without rhabdomyolysis (*n* = 36).

	Total (*n* = 36)	Rhabdomyolysis (*n* = 17)	Not rhabdomyolysis (*n* = 19)	*P* value
*Demographic data*				
Age (years)	48 (30–65)	35 (23–51)	57 (44–68)	0.01
Male gender	31 (86)	17 (100)	14 (74)	0.02
SAPS II score	41 (32–48)	38 (31–48)	41 (32–49)	0.90
ISS score	34 (26–47)	43 (17–50)	34 (27–41)	0.24
*Indication for CRRT *				
Fluid overload	19 (53)	8 (47)	11 (58)	0.52
Hyperkalemia	20 (56)	9 (53)	11 (58)	0.77
Acidosis	15 (42)	3 (18)	12 (63)	0.01
Uremia	9 (25)	4 (24)	5 (26)	0.85
Rhabdomyolysis	17 (47)	17 (100)	0 (0)	<0.01
*Kidney function at start of CRRT*				
Diuresis (mL/day)	536 (256–1692)	522 (252–2060)	550 (255–1410)	0.80
Serum potassium (mmol/L)	5.0 (4.2–5.5)	4.4 (4.0–5.7)	5.1 (4.8–5.5)	0.20
Serum urea (mmol/L)	17.8 (12.5–28.4)	13.6 (10.6–25.0)	22.2 (16.3–29.8)	0.07
Serum creatinine (*μ*mol/L)	249 (204–376)	247 (198–483)	269 (215–361)	0.95
Serum creatine kinase (U/L)	4638 (460–24414)	24415 (9660–45740)	603 (135–2152)	<0.01
*CRRT performance data*				
Median daily CRRT duration (hours)	18.6 (14.9–21.3)	16.3 (12.4–20.0)	19.7 (15.6–21.8)	0.19
Median Δurea	-0.05 (−0.12–0.09)	-0.06 (−0.19–0.03)	-0.05 (−0.08–0.11)	0.20
Median Δcreatinine	0.05 (−0.04–0.17)	0.03 (−0.17–0.15)	0.12 (0.01–0.17)	0.21
Filter clotting pauses	68 (54)	36 (55)	32 (53)	0.90
Therapeutic pauses	32 (25)	25 (38)	7 (12)	<0.01
Catheter-related pauses	12 (10)	3 (5)	9 (15)	0.05
Filter timeout pauses	7 (6)	1 (2)	6 (10)	0.04
Diagnostic pauses	7 (6)	5 (8)	2 (3)	0.30
*Outcome*				
Dialysis-dependent 3 months	0 (0)	0 (0)	0 (0)	n.c.
Dialysis-dependent 1 year	0 (0)	0 (0)	0 (0)	n.c.
Mortality 3 months	11 (31)	3 (18)	8 (42)	0.11
Mortality 1 year	13 (36)	4 (24)	9 (47)	0.14

Categorical data are presented as number (percent) and compared using Pearson chi-square test. Continuous data are expressed as median (interquartile range) and are compared using 2-tailed Mann-Whitney *U* test. SAPS II: simplified acute physiology score II. ISS: injury severity score. n.c.: not calculated. See text for other definitions.

**Table 2:** Individual data: Trauma patients with acute kidney injury with continuous renal replacement therapy (CRRT) grouped with and without rhabdomyolysis (*n* = 36).

Patient number	Rhabdomyolysis	CRRT days	Median daily CRRT duration	Median Δurea	Median Δcreatinine
1	No	5	21,1	−0,05	−0,05
2	No	5	19,7	0,10	0,22
3	No	5	22,3	0,15	0,15
4	No	5	22,3	0,12	0,17
5	Yes	5	16,8	0,03	0,14
6	Yes	5	20,8	0,01	0,01
7	No	3	18,7	−0,10	0,16
8	Yes	5	22,8	0,03	0,04
9	No	5	18,7	0,11	0,28
10	No	5	21,8	−0,08	0,02
11	Yes	5	19,2	0,23	0,25
12	No	4	16,1	−0,01	0,13
13	Yes	1	9,1		
14	No	5	23,8	−0,14	0,01
15	Yes	4	16,1	0,17	0,18
16	No	5	9,8	−0,23	−0,12
17	Yes	5	10,3	−0,18	−0,02
18	No	5	21,4	0,08	0,03
19	Yes	4	18,5	−0,14	−0,18
20	Yes	4	15,8	−0,02	0,21
21	Yes	1	24,0		
22	No	2	15,6	−0,06	0,05
23	Yes	5	16,3	−0,20	−0,24
24	No	5	23,5	0,17	0,25
25	Yes	5	18,5	−0,10	−0,05
26	No	4	11,5	−0,08	−0,15
27	Yes	2	16,1	0,03	0,08
28	No	5	14,4	−0,09	−0,03
29	No	5	19,4	0,04	0,01
30	No	4	14,6	−0,07	0,18
31	Yes	5	13,0	−0,30	−0,32
32	Yes	1	24,0		
33	No	5	19,9	0,18	0,12
34	Yes	3	11,8	−0,35	0,05
35	No	5	20,9	−0,07	0,12
36	Yes	3	8,6	−0,19	−0,17

Δurea = (Urea_day N_−Urea_day N + 1_)/Urea_day N_ where Urea_day N_ is the serum concentration of urea at day N, and Urea _day N + 1 _ is the serum concentration of urea at day N+1.

Δcreatinine = (Creatinine_day N_−Creatinine_day N + 1_)/Creatinine_day N_ where Creatinine_day N_ is the serum concentration of creatinine at day N, and Creatinine_day N + 1_ is the serum concentration of creatinine at day N+1.

## References

[1] Piccinni P, Cruz DN, Gramaticopolo S, Garzotto F, Dal Santo M, Aneloni G (2011). Prospective multicenter study on epidemiology of acute kidney injury in the ICU: a critical care nephrology Italian collaborative effort (NEFROINT). *Minerva Anestesiologica*.

[2] Bagshaw SM (2006). The long-term outcome after acute renal failure. *Current Opinion in Critical Care*.

[3] McCunn M, Reynolds HN, Reuter J, McQuillan K, McCourt T, Stein D (2006). Continuous renal replacement therapy in patients following traumatic injury. *International Journal of Artificial Organs*.

[4] Bagshaw SM, Berthiaume LR, Delaney A, Bellomo R (2008). Continuous versus intermittent renal replacement therapy for critically ill patients with acute kidney injury: a meta-analysis. *Critical Care Medicine*.

[5] Ghahramani N, Shadrou S, Hollenbeak C (2008). A systematic review of continuous renal replacement therapy and intermittent haemodialysis in management of patients with acute renal failure. *Nephrology*.

[6] Rabindranath K, Adams J, MacLeod AM, Muirhead N (2007). Intermittent versus continuous renal replacement therapy for acute renal failure in adults. *Cochrane Database of Systematic Reviews*.

[7] Pannu N, Klarenbach S, Wiebe N, Manns B, Tonelli M (2008). Renal replacement therapy in patients with acute renal failure: a systematic review. *Journal of the American Medical Association*.

[8] Zarbock A, Singbartl K, Kellum JA (2009). Evidence-based renal replacement therapy for acute kidney injury. *Minerva Anestesiologica*.

[9] Seabra VF, Balk EM, Liangos O, Sosa MA, Cendoroglo M, Jaber BL (2008). Timing of renal replacement therapy initiation in acute renal failure: a meta-analysis. *American Journal of Kidney Diseases*.

[10] Palevsky PM (2009). Intensity of continuous renal replacement therapy in acute kidney injury. *Seminars in Dialysis*.

[11] Van Wert R, Friedrich JO, Scales DC, Wald R, Adhikari NKJ (2010). High-dose renal replacement therapy for acute kidney injury: systematic review and meta-analysis. *Critical Care Medicine*.

[12] Fealy N, Baldwin I, Bellomo R (2002). The effect of circuit “down-time” on uraemic control during continuous veno-venous haemofiltration. *Critical Care and Resuscitation*.

[13] Uchino S, Fealy N, Baldwin I, Morimatsu H, Bellomo R (2003). Continuous is not continuous: the incidence and impact of circuit “down-time” on uraemic control during continuous veno-venous haemofiltration. *Intensive Care Medicine*.

[14] Vesconi S, Cruz DN, Fumagalli R (2009). Delivered dose of renal replacement therapy and mortality in critically ill patients with acute kidney injury. *Critical Care*.

[15] Pollinder S, Haagsma JA, Toet H, Brugmans MJ, van Beeck EF (2010). EUROCOST and APOLLO reference groups. Burden of injury in childhood and adolescence in 8 European countries. *BMC Public Health*.

[16] Uchino S, Kellum JA, Bellomo R (2005). Acute renal failure in critically ill patients: a multinational, multicenter study. *Journal of the American Medical Association*.

[17] Vivino G, Antonelli M, Moro ML (1998). Risk factors for acute renal failure in trauma patients. *Intensive Care Medicine*.

[18] Holt SG, Moore KP (2001). Pathogenesis and treatment of renal dysfunction in rhabdomyolysis. *Intensive Care Medicine*.

[19] Beitland S, Moen H, Os I (2010). Acute kidney injury with renal replacement therapy in trauma patients. *Acta Anaesthesiologica Scandinavica*.

[20] Le Gall JR, Lemeshow S, Saulnier F (1993). A new Simplified Acute Physiology Score (SAPS II) based on a European/North American multicenter study. *Journal of the American Medical Association*.

[21] Baker SP, O’Neill B, Haddon W, Long WB (1974). The injury severity score: a method for describing patients with multiple injuries and evaluating emergency care. *Journal of Trauma*.

[22] Huerta-Alardín AL, Varon J, Marik PE (2005). Bench-to-bedside review: rhabdomyolysis—an overview for clinicians. *Critical Care*.

[23] Plank LD, Hill GL (2000). Sequential metabolic changes following induction of systemic inflammatory response in patients with severe sepsis or major blunt trauma. *World Journal of Surgery*.

[24] Oudemans-van Straaten HM (2010). Citrate anticoagulation for continuous renal replacement therapy in the critically ill. *Blood Purification*.

[25] Cirocchi R, Abraha I, Montedori A (2010). Damage control surgery for abdominal trauma.. *Cochrane Database of Systematic Reviews*.

[26] Dormagen JB, Totterman A, Roise O, Sandvik L, Klow NE (2010). Efficacy of plain radiography and computer tomography in localizing the site of pelvic arterial bleeding in trauma patients. *Acta Radiologica*.

[27] Gaarder C, Dormagen JB, Eken T (2006). Nonoperative management of splenic injuries: improved results with angioembolization. *Journal of Trauma*.

[28] Gaarder C, Naess PA, Eken T (2007). Liver injuries-Improved results with a formal protocol including angiography. *Injury*.

[29] Townsend DR, Bagshaw SM, Jacka MJ, Bigam D, Cave D, Gibney RTN (2009). Intraoperative renal support during liver transplantation. *Liver Transplantation*.

[30] Bellomo R (2011). Acute kidney injury: the Italian perspective. *Minerva Anestesiologica*.

[31] Liangos O, Rao M, Ruthazer R (2004). Factors associated with urea reduction ratio in acute renal failure. *Artificial Organs*.

[32] Vanholder R, De Smet R, Glorieux G (2003). Review on uremic toxins: classification, concentration, and interindividual variability. *Kidney International*.

[33] Gatward JJ, Gibbon GJ, Wrathall G, Padkin A (2008). Renal replacement therapy for acute renal failure: a survey of practice in adult intensive care units in the United Kingdom. *Anaesthesia*.

[34] Uchino S, Bellomo R, Morimatsu H (2007). Continuous renal replacement therapy: a worldwide practice survey: the beginning and ending supportive therapy for the kidney (B.E.S.T. Kidney) investigators. *Intensive Care Medicine*.

[35] Bellomo R, Cass A, Cole L, Finfer S, Gallagher M, Goldsmith D (2008). Renal replacement therapy for acute kidney injury in Australian and New Zealand intensive care units: a practice survey. *Critical Care and Resuscitation*.

